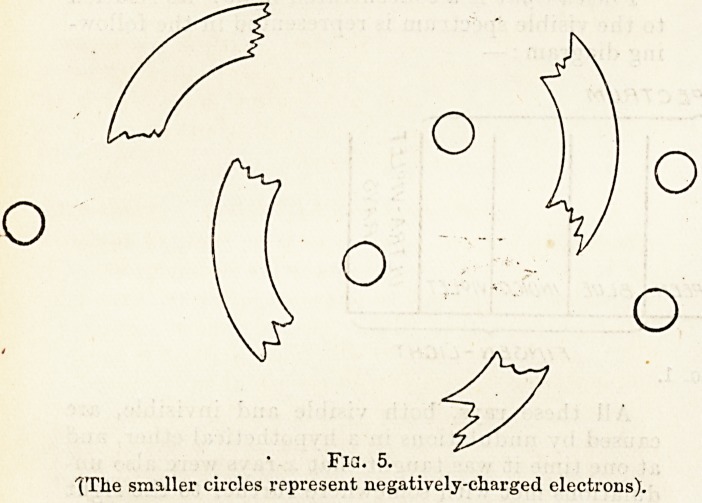# The Present Position of Radiation in Treatment

**Published:** 1906-06-02

**Authors:** Gerald Sichel

**Affiliations:** Surgeon-in-charge of the Actino-therapeutic Department, Guy's Hospital.


### HOSPITAL CLINICS.

### THE PRESENT POSITION OF RADIATION IN TREATMENT.

By GERALD SICHEL, F.R.C.S., F.C.S., Surgeon-in-charge of the Actino-therapeutic Department, Guy's Hospital.

### I.—GENERAL CONSIDERATIONS.

RADIOTHERAPY as a practical measure dates from 1893, when Finsen proposed the red light treatment for small-pox, and 1896, when it was observed that *x*-rays were capable of giving rise to a dermatitis followed by loss of hair over the affected area; and, in consequence, Freund decided to make use of this property in treating a hairy mole.

Like all other new forms of treatment, radiation has been taken up by the indiscriminating lay press, and it says much for its value that it has survived the ordeal and still remains a recognised method of treatment.

From being vaunted as the magic panacea for all evils under the sun, radiotherapy still remains as an extremely valuable remedy in a limited field. Scientifically as well as medically but little is known of the various agencies employed under the term radiation.

I propose here to shortly deal with

1. Finsen light.
2. *x*-rays.
3. Radium emanations.
4. High frequency electricity.

Finsen began his great work by observing that small-pox cases from which the chemical rays of the solar spectrum were excluded, did better than those that were exposed to them: he finally discovered that these very chemical rays, which were harmful in small-pox, were beneficial in cases of lupus.

*Finsen light* is a concentrated light: its relation to the visible spectrum is represented in the following diagram:—

FIG. 1.

All these rays, both visible and invisible, are caused by undulations in a hypothetical ether, and at one time it was taught that *x*-rays were also undulations met with somewhere further to the right than the ultra-violet waves.

More recent research and opinion on *x-rays* has gradually constructed another theory, which practically requires a mind capable of conceiving movement without matter.

In other words, electricity is a *condition* of matter, but the latest theory demands that we should picture in our mind's eye infinitely minute particles of electricity—smaller by far than chemical atoms—which are called "electrons."

FIG. 2.

FIG. 3.

FIG. 4.

Both cathodal rays and *x*-rays must be looked upon as something "particulate." One method of mentally picturing the *x*-rays has been as follows: The cathodal rays are considered analogous to a shower of bullets bombarding any obstacle in their path, the result being *x*-rays, which are comparable to the reports caused by such a shower of bullets on a target.

Reverting to the electron theory, *x*-rays have been described as a collection of "negatively"

charged electrons surrounded by a "positive" envelope; if in their passage they come into collision with any substance, some of the "negative" electrons escape, and the "positive" envelope still speeds on containing fewer electrons; this happens time after time until finally the "positive" envelope itself becomes totally disrupted and free negative electrons and free fragments of "positive" envelope career along.

Fig. 5.
(The smaller circles represent negatively-charged electrons).

Now which of these conditions is responsible for the results of *x*-ray treatment?

Nobody at present knows; in other words, we are dealing with a powerful therapeutic agent of which we practically know nothing. And our difficulties are multiplied by the fact that, besides *x*-rays, the *x*-ray tube produces heat, ozone, cathodal rays, ultra-violet rays, and probably rays of totally unknown character.

Which of these is really the active agent?

Which causes dermatitis, for instance?

*Radium* emanations have a useful but decidedly small field in therapeutics.

Radium rays have been classified as Alpha, Beta, and Gamma rays; the Alpha rays eventually changing into helium, the Beta being similar to or identical with cathodal rays, and the Gamma similar to or identical with *x*-rays.

What do we know of helium as a curative agent? Is it to the Alpha or Beta or Gamma rays that we have to look for our therapeutic results? Again we are obliged to admit we do not know.

*High frequency currents.*—I am not sure that they should not be described as charges, *not* currents—have been in the field as a therapeutic agent for the past quarter of a century.

High frequency electricity as applied medically is characterised by immense voltage, but practically the current alternates so rapidly—*with such high frequency, in fact*—that there is not time for any dangerous or deleterious effect to take place, even if pretty strong currents are used.

I have in the above as shortly as possible attempted to explain the physical properties of the various agents used in radiotherapy, as at present accepted. Before entering into details I shall continue to generalise but from two other points of view. I have considered the physical; now I propose to take the utilitarian, and finally the commercial, aspect of the position.

From a utilitarian point of view it must be realised that 90 per cent. of the cases sent to a department such as mine have been given up as utterly hopeless in other fields of treatment. Recurrent carcinoma mammæ, inoperable carcinoma of the cervix, hopelessly recurrent cancer in the glands or elsewhere—these form a goodly proportion of the cases which are sent to radiotherapists, and by which, if statistics were everything, strictly speaking, radiotherapy should stand or fall. I have had many dozens such to treat with *x*-rays, and my experience agrees with most other workers in the same field. The majority of cases fail to receive the slightest benefit; in a fair number (if the patients themselves are to be believed) the pain is somewhat eased—sometimes considerably: in a very few I think the rate of growth seems to have been retarded. I have heard of one or two cases, and seen the photograph of one case diagnosed as malignant, which have—at all events for the time—been cured.

I have entirely failed to do any good in cases of ordinary sarcoma, but I have only had one or two. On the other hand, it is well known that such cases as mycosis fungoides, sarcomatosis cutis, and Kaposi's disease are materially improved, and even apparently cured by *x*-rays. These are all rare diseases; but to cure even one case of otherwise certainly fatal termination is, I think, a great triumph.

It leads one to hope that possibly we may, after all, be able to attack cancer successfully; possibly radiotherapy, combined with serotherapy, may supply the much desired result. At the present time *x*-rays are coming to be looked upon as *the* treatment for rodent ulcer, ringworm, and certain cases of lupus and scrofulodermia.

Finsen light is generally the most successful treatment for lupus. The ordinary surgical treatment of lupus vulgaris—short of complete excision of small spots—has never been satisfactory; scraping, scarification, and so forth leave the disfiguring scars which are only too well known to all of us, and the disease more often than not returns.

It looks possibly as if here also radiotherapy may combine with serotherapy, and that perhaps the most successful treatment will be secured by using Finsen light together with Wright's vaccine method.

Radium treatment is, after a great flourish of trumpets, now but little used. I think that perhaps the pendulum has almost swung too far the other way, as although a much weaker agent than *x*-rays, in its weakness lies its strength, inasmuch as it can be safely left with an intelligent patient to use at his own house, in cases of small rodent ulcer, small spots of lupus or small moles, where even a minor operation is not desired; at the same time I hardly think that radium salts are really worth, for purely therapeutic purposes, the high price they command in the market.

Finally, high frequency electricity. I hope I shall not be considered unduly pessimistic, but it must be conceded on all sides that the very great expectations raised by this form of treatment have not been justified.

It is an undoubted fact that a treatment of 15 minutes or so as a general rule raises the patient's temperature a degree or two; but it is not difficult to raise the temperature to this extent in persons with a highly strung nervous temperament, and it is chiefly in neuroses and skin diseases that this treatment has been exploited. As I am at present only generalising it will suffice to say that along with others I have been disappointed with high frequency.

At the same time it cannot be said that any finality has been reached with this or any other form of radiotherapy.

With regard to the commercial aspect of the position, I have purposely set myself a very difficult and delicate task.

In the first place, radiotherapy has been up to the present to a great extent in the hands of—medically speaking—unqualified persons; in the second the great hopes falsely raised for it by the lay press have —I am afraid in some directions justly—left the stigma of the quack and the charlatan on the whole treatment; in the third place, as I have said before, 90 per cent. of the cases coming up for treatment have been given up as absolutely hopeless in other fields of therapeutics, and finally a complete outfit is an extremely expensive business.

Taking these four points into consideration, and the fact that after all in many fields radiotherapy is only on its trial, it seems to me more than a pity that in London at least an institution is not established for the carrying out of the treatment and the scientific study of the various agents employed in radiotherapy.

There is a vast amount of good which can, we now know, be accomplished; but so far as statistics are concerned the wheat has to be sifted from the chaff. At present it seems to me that organisation is what is needed.

For the individual practitioner to undergo the great expense of an entire outfit, nurses' charges, and the unprofitable labour of having to treat 100 cases with the prospect of, say, 30 cures, requires considerable capital, and more than considerable enthusiasm and self-confidence; and even then probably but little opportunity for experimental work would be forthcoming.

I do not mean—it would be an impertinence if I did—to belittle the work at present being done by such men as Lewis-Jones, Sequeira, Macleod, Adamson, Morton, Lister, and others; but I do mean to say that individual workers are struggling against very adverse circumstances. There is proof of so much good in radiotherapy, and so much research work is necessary, that it would be well worth the trouble of some responsible body to appoint a committee to investigate the means of getting the most that can be got out of rays and electricity generally.

It is certain that no private individual can be expected to launch forth on his own at great expense, and merely as an experimental scientist. I believe that some years ago Mr. Edmund Owen suggested a central general electrical and radiotherapeutic institute in London. I would again advocate most strongly such a scheme.

The application of all radiotherapeutic measures should, I think, be under immediate medical supervision, but should actually be made by a nurse.

The nurse, of course, requires special training and instruction, but the carrying out of the details is decidedly a nurse's, and not a medical man's duty.

Qualified medical supervision is, however, not only necessary, but if this should not be forthcoming, its absence would be used, and rightly too, as an argument that entirely uncertificated persons are fitted to undertake the responsible charge of the patients.

Medicine and pure science, pathology and physics, might, with probably great benefit to mankind, combine, and put radiotherapy on a thoroughly certain, safe, sound, and scientific basis.

(*To be continued.*)

## Figures and Tables

**Fig. 1. f1:**
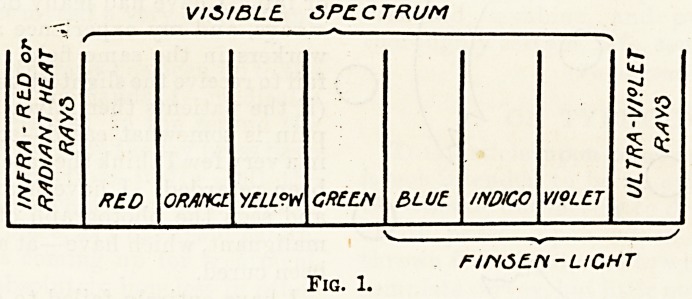


**Fig. 2. f2:**
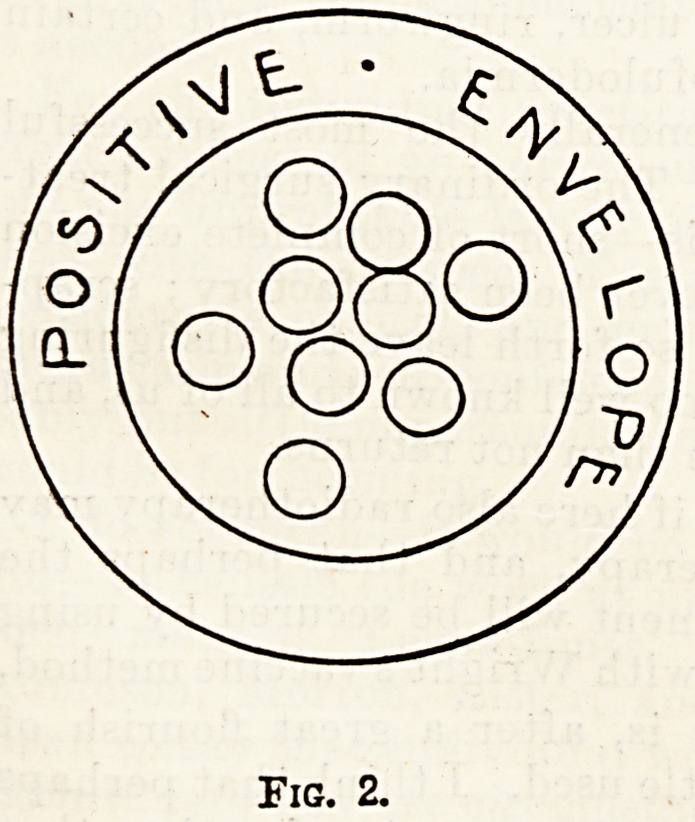


**Fig. 3. f3:**
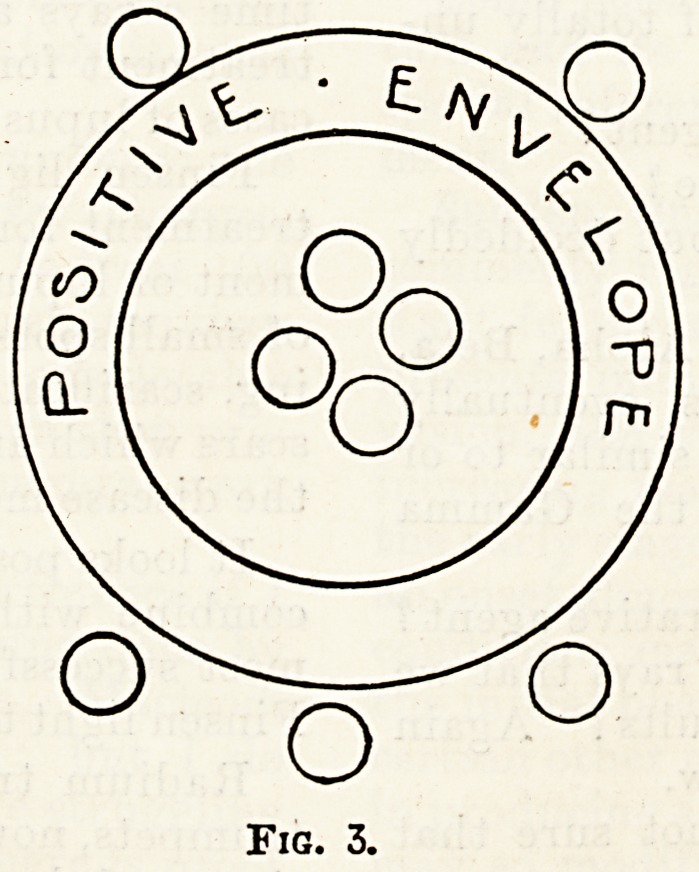


**Fig. 4. f4:**
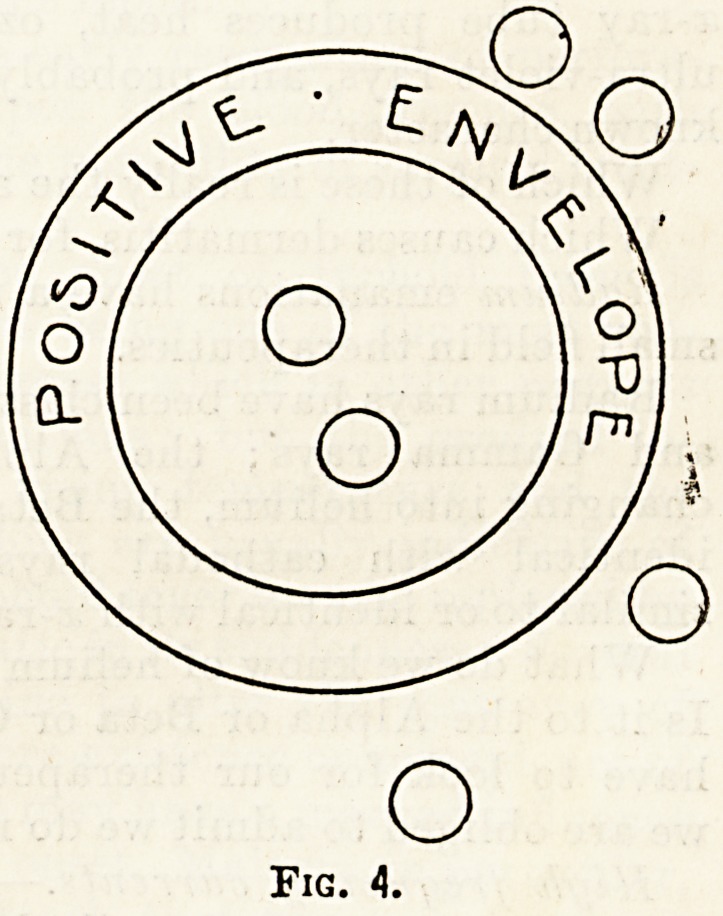


**Fig. 5. f5:**